# PEG-fusion of viable sciatic nerve isografts restores axonal structure and behavioral recovery after segmental-loss sciatic nerve injuries in Lewis rats

**DOI:** 10.1371/journal.pone.0349204

**Published:** 2026-05-21

**Authors:** Cathy Z. Yang, Liwen Zhou, Alexander M. Schafer, Alexa N. Olivarez, Guhan Periyasamy, Varun Gokhale, Rhea Sood, Henry A. Garcia, Arjun Agarwal, Joseph F. Alderete, George D. Bittner

**Affiliations:** 1 Department of Neuroscience, University of Texas at Austin, Austin, Texas, United States of America; 2 Department of Orthopedics, University of Texas Health Science Center at San Antonio, San Antonio, Texas, United States of America; Advanced Materials Technology Research Institute, National Research Centre, EGYPT

## Abstract

Segmental-loss peripheral nerve injuries (SL-PNIs) often produce severe deficits in sensory/motor functions and voluntary behaviors. The current gold standard for repair is neurorrhaphy of a cable autograft that (1) produces donor site morbidity; (2) results in rapid Wallerian degeneration (WD) of severed distal nerve segments; (3) relies on slow (1–2 mm/day) axonal regeneration; (4) does not prevent atrophy of denervated muscles and sensory structures; and (5) results in poor to non-existent recovery of sensory/motor functions and voluntary behaviors, especially with longer segmental-loss gaps and/or with denervated targets located rather distal to a proximal SL-PNI. Our study used genetically identical Lewis rats as a model system for isograft transplants. Neurorrhaphy of viable peripheral nerve isografts (VPNIs) of 5- or 10-mm length was performed to repair sciatic SL-PNIs of 4- or 8-mm gap length, respectively. Animals were repaired with a set of well-specified solutions that did (PEG-fusion group) or did not (Negative Control [NC] group) contain 50% w/w 3.35 kDa polyethylene glycol (PEG), an axolemmal fusogen at that specific weight and concentration. We also examined the effects of locally applied FK506 on 10-mm VPNI repairs both with and without PEG-fusion. We hypothesized that PEG-fusion groups would show better axonal morphology and behavioral recovery, as assessed by the Sciatic Functional Index (SFI), compared to NC groups regardless of the gap length. We also hypothesized that FK506 would improve the effects of PEG-fusion by reducing inflammation. Our data showed that PEG-fused VPNI groups had significantly larger axonal diameters and lower g-ratios, less WD, and better SFI scores compared to NC groups regardless of the gap lengths for SL-PNIs. However, localized FK506 treatment only transiently improved axonal regeneration and impaired long-term SFI behavioral recovery, which is the most important measure of successful repairs. In conclusion, PEG-fusion repair technologies show great potential for improving clinical treatments.

## Introduction

Segmental-loss peripheral nerve injuries (SL-PNIs) result in immediate loss of sensorimotor function, denervation of muscles and sensory organs, and Wallerian degeneration (WD) of nerve axons distal to the injury [1–4]. Current experimental and clinical procedures to repair SL-PNIs involve microsuturing donor bridging materials to severed host nerve ends (neurorrhaphy) to promote axonal regeneration [4]. However, this axonal regeneration is non-specific and slow (1–2 mm/day), and behavioral recovery is often poor or absent, particularly for SL-PNIs with larger gap defects (≥5 mm in rats, ≥ 5 cm in humans) or longer distances between the injury site and denervated distal targets [5–8].

Polyethylene glycol (PEG)-fusion has recently emerged as a promising alternative repair strategy both in experimental animal models and clinical case studies [6,9–25]. PEG-fusion technologies employ a series of bioengineered solutions, including 50% w/w 3.35 kDa PEG, to non-specifically fuse the severed open ends of closely apposed donor and host axons. In preclinical rat models of SL-PNIs, PEG-fusion repairs using viable peripheral nerve allografts (i.e., axons conduct action potentials and glial and other supporting cells are viable) restore axonal continuity, prevent WD of approximately 40–60% of axons that successfully PEG-fuse, preserve neuromuscular junction innervation, and restore voluntary behaviors within weeks to months [16,19–25].

However, minimal data have been published for PEG-fused viable peripheral nerve autografts in rats [24,26] or PEG-fused viable peripheral nerve isografts (VPNIs) in any experimental model. VPNIs are valuable models for drug testing because, unlike allografts, they do not show pronounced immune responses or rejection. Compared to autografts, VPNIs of appropriate diameters and lengths can also be more easily harvested, avoiding cabling, nerve stretching, and donor site morbidity in the host. Therefore, in this study, we assessed the efficacy of PEG-fusion to repair SL-PNIs using VPNIs in rats.

Tacrolimus (FK506) is an FDA-approved immunosuppressant [27,28]. Although favored for ease of administration, systemic FK506 administration is limited by its neurotoxicity [29–32]. When used locally in peripheral nerve autografts or allografts, FK506 is a potent neuromodulator that preserves the graft’s structure, minimizes scarring, supports Schwann cells, and creates a more supportive environment for regenerating axons by reducing inflammation—leading to better functional recovery [27,33–40].

We hypothesized that PEG-fusion repair using VPNIs would produce behavioral recovery and that localized FK506 delivery would further improve behavioral outcomes by promoting regeneration of axons that were not successfully repaired at the time of PEG-fusion. We performed PEG-fusion and Negative Control (NC; neurorrhaphy only) repairs in inbred Lewis rats using 5-mm VPNIs (for ~4-mm gap defects), 10-mm VPNIs (for ~8-mm gap defects), and 10-mm VPNIs with localized FK506 delivered in TISSEEL fibrin gel that reportedly facilitates mechanical stability and may decrease hemorrhaging [41–43]. Successful PEG-fusion repair was confirmed by electrophysiological testing within minutes after the repair. Axonal morphology was examined at 3 and 6 weeks post-operatively (PO). Behavioral recovery was assessed weekly by the Sciatic Functional Index (SFI) test. Our results showed that PEG-fusion produced SFI recovery across both shorter and longer gap defects, but local delivery of FK506 produced only transient regenerative benefits and ultimately impaired long-term behavioral recovery in rats repaired with PEG-fused VPNIs.

## Materials and methods

### Animals

All experimental procedures adhered to the *National Institutes of Health Guide for the Care and Use of Laboratory Animals* (8th ed., National Research Council, 2011) and were approved by the Institutional Animal Care and Use Committee (IACUC) at The University of Texas at Austin (AUP20252200278, approved 06/09/2025). Male (350–500 g) and female (225–300 g) inbred Lewis rats aged 3–12 months were housed 2–3/cage and maintained on a 12-hour light/dark cycle with food and water given ad libitum. A total of 126 Lewis rats were used in this study. Surgical and behavioral procedures were performed during the light cycle. Early humane endpoints were determined by complications such as tumors, severe weight loss, and self-mutilation of digits. No rat met these criteria in this study. All rats reached the end of experiments and were euthanized within 1–3 days.

### Surgical procedures

Surgical procedures were performed as previously described [19,20,23]. Briefly, host Lewis rats were anesthetized with isoflurane (4% induction, 2% maintenance)/oxygen mixture at 1.5 L/min. Rats received subcutaneous injections of lidocaine along the incision site prior to incision. The lateral aspect of the left hindlimb was shaved and sterilized, and a 2−3 cm incision was made through the skin and the biceps femoris to expose the sciatic nerve. The sciatic nerve was ablated for the desired length (~4-mm gap for 5-mm graft, ~ 8-mm gap for 10-mm graft) at the mid-thigh level by fine dissection scissors in a Ca^2+^-containing isotonic extracellular solution (0.9% NaCl with 2 mM CaCl_2_). In both PEG-fusion and NC groups, severed sciatic nerve stumps were irrigated with 0.5% methylene blue followed by diluted Normosol-R. Size- and sex-matching donor Lewis VPNIs were freshly harvested and trimmed to approximate the gap length. Both ends of the host sciatic nerve stumps were trimmed flush to enable their close apposition to VPNIs with at least four 10−0 microsutures through the epineurium and/or perineurium sheaths. For PEG-fused rats, lesion sites were then submerged in a sterile solution of 50% w/w 3.35 kDa PEG (Sigma-Aldrich, St. Louis, MO, USA) in distilled water for 1–2 minutes to non-specifically fuse the closely apposed, open axonal ends. NC rats underwent neurorrhaphy, but were not treated with PEG. Following neurorrhaphy, lesion sites in both PEG-fused and NC rats were flushed several times with a Ca^2+^-containing isotonic extracellular solution to accelerate Ca^2+^-induced vesicle accumulation and seal any remaining open axons [44].

*In vivo* compound action potentials (CAPs) were confirmed as previously described [20,23]. Briefly, *in vivo* CAPs were recorded immediately following PEG-fusion to confirm electrophysiological continuity in all surgeries. Stimulating and recording electrodes were placed proximally and distally to the VPNI at a distance of ≥ 1 cm. Sciatic nerves were stimulated at 1 V using 0.1 ms square wave depolarizations at 1 Hz with a 0.1 ms delay from the trigger signal using a PowerLab 4/35 (AD instruments, Sydney, Australia) and recorded using a Dual Bio Amp (AD instruments, Sydney, Australia).

Following CAP recording, FK506 (Fisher Scientific, Hampton, NH, USA) in TISSEEL^®^ (Baxter, Deerfield, IL, USA) was applied to the surgical site to a group of rats. For FK506 loading, solid FK506 powder was resuspended in ethanol at 200 mg/mL before it was mixed into a thrombin solution for a final concentration of 2 mg/mL, which became 1 mg/mL in the final fibrin gel after mixing. A sufficient volume of fibrin gel was applied to surround the repaired nerve (typically ~0.4 mL for males and ~0.2 mL for females). The *in vivo* biodistribution of TISSEEL-delivered FK506 at similar doses has been well-characterized in previous studies [34,36]: FK506 remained mainly in the nervous tissues and surrounding gluteal muscle, and was undetectable in the blood. No animal in the current study exhibited significant weight loss or other signs of systemic neurotoxicity from FK506 following operations.

The muscle incision was closed with 5−0 sutures, and the skin was closed with wound clips. Rats recovered from surgery on heating pads before being returned to standard housing. Rats received 5 mg/kg subcutaneous injections of carprofen during surgery and PO daily for the next three days.

### Sciatic Functional Index (SFI) tests

The SFI as a measure of voluntary behavior involving sensory and motor functions was performed as previously described [20,45]. Prior to surgical operations, rats were trained to become acclimated to the testing apparatus and procedure. SFI tests were performed and scored by testers blinded to the surgical groups for up to 6 weeks PO. Rat hind paws were marked with red (unoperated limb) and blue ink (operated limb). Rats were placed on one end of a slightly inclined board (1.52 m long, 10.2 cm wide) lined with paper strips and allowed to run back to the home cage on the other end. Inked paw prints were analyzed for paw length, total toe spread, and intermediate toe spread to compute SFI scores. A successful SFI trial consisted of three consecutive steps by each hindlimb without hesitating or stopping. Two trials were obtained from each rat to compute the average score for each PO time point. Unoperated rats had SFI scores of 0 ± 30, i.e., had gait symmetry. Impaired movement of the injured hind paw produced more-negative SFI scores.

### Morphological analyses

Nerve samples were fixed and embedded as previously described [24]. Briefly, samples were fixed in 2% paraformaldehyde/3% glutaraldehyde in 0.1 M sodium cacodylate buffer. 2–3 mm of the proximal, mid-VPNI, and distal segments, each 1–2 mm away from the suture sites, were harvested. All samples were post-fixed in 1% osmium tetroxide/1% potassium ferrocyanide followed by 1% aqueous uranyl acetate prior to dehydration and embedding in Hard Plus Resin 812 (Electron Microscopy Sciences, Hatfield, PA, USA). Samples were incubated at 60°C for 48–72 hours prior to sectioning. Glass knife thick sections (0.5 μm) were obtained using a Leica ultramicrotome, stained with toluidine blue, and imaged on a Zeiss Axiovert 200M fluorescent light microscope with an HR3 camera (Hebron, KY, USA). Axon density, diameter, and g-ratio were analyzed using ImageJ (version 1.53m, National Institutes of Health, Bethesda, MD, USA) on randomly chosen regions of interest (ROIs). For axon density, 5 ROIs were analyzed per animal in each surgical group. Generally, > 200 axons were analyzed in PEG-fused samples. As few as 10 axons were analyzed in some ROIs in NC samples (3w PO) that had very few surviving axons. For axon diameter and g-ratio at 6w PO, 150–200 axons from at least 3 ROIs per animal were analyzed in each surgical group. For proximal segments, which served as controls, 1 animal was analyzed in each surgical group. For mid-VPNI and distal segments, 2 animals were analyzed in each surgical group, except for the 10-mm PEG-fused VPNI+FK506 group where only one animal that had successful SFI recovery was analyzed. Therefore, double the number of ROIs (10 ROIs) and axons (>300 axons) were quantified from this one sample for this group’s morphological analyses.

### Statistical analyses

All statistical analyses were performed using GraphPad Prism (version 8, GraphPad Software, Boston, MA, USA, www.graphpad.com). The sample sizes were determined *a priori* using G*Power analysis software at p < 0.05 and power at 0.8, with 10–12 animals/group for weekly SFI data analyses (Repeated-measures ANOVA test, partial η^2^ = 0.05). Data from male and female rats were pooled for both axon morphology and SFI, as previous studies reported no sex-related differences [19,20,45]. Assumptions of normality were assessed using Shapiro-Wilk tests. Morphological data were analyzed by ordinary one-way analysis of variance (ANOVA) followed by *post hoc* Tukey’s multiple comparison tests. SFI data were analyzed by two-way ANOVA with repeated measures followed by *post hoc* Tukey’s multiple comparison tests. The success rates of PEG-fusion surgeries were analyzed by chi-square test. A 95% confidence interval was used. All data are presented as mean ± standard error of the mean (SEM). Animal/axon n numbers are shown in each figure panel or legend.

## Results

### PEG-fused VPNI repairs of SL-PNIs of different gap lengths produce similar SFI recovery

**Fig 1:**
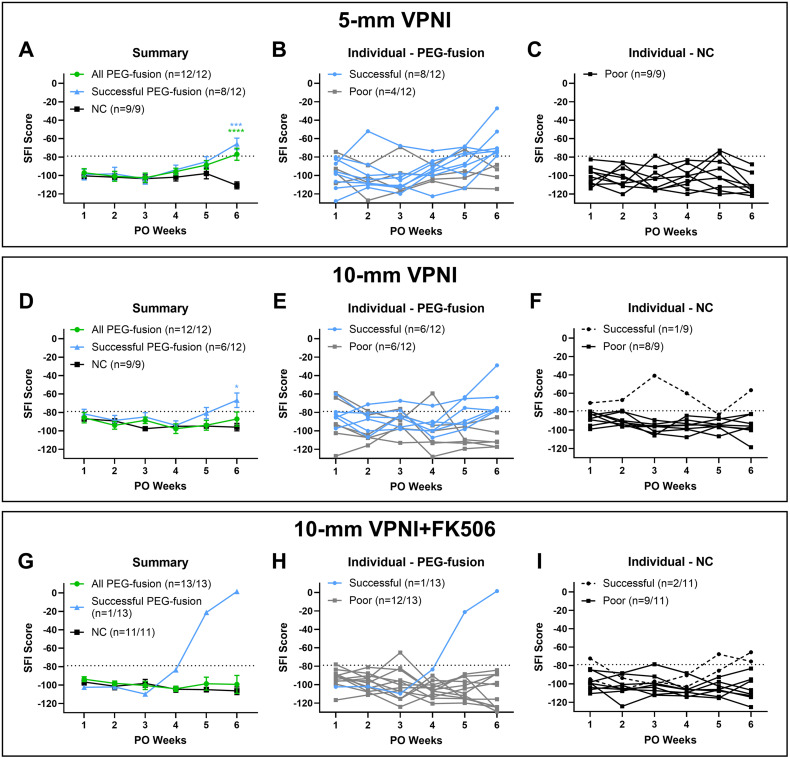
PEG-fusion produces SFI recovery across both VPNI lengths, but success is reduced with local FK506 delivery. Successful and poor SFI recovery were defined as maintaining SFI score of>-79 or failing to reach SFI score of −79 by 6 weeks PO, respectively. (**A-C**) 5-mm VPNI repairs. (**D-F**) 10-mm VPNI repairs. (**G-I**) 10-mm VPNI+FK506 repairs. Left column: Summary data of all PEG-fusion rats (green) vs. all NC rats (black). Successful PEG-fused rats (blue) were separately plotted. Middle column: Individual data of Successful and Poor PEG-fused rats. Right column: Individual data of Successful and Poor NC rats. *s indicate Tukey’s multiple comparisons test results. *: *p* < 0.05, ***: *p* < 0.001, ****: *p* < 0.0001. Values are means ± SEM in summary panels. Sample sizes (n) are listed in the keys in each figure panel.

As described in Methods, we generated the following four groups of rats having SL-PNI gap lengths of 4- or 8-mm repaired by VPNIs of slightly longer lengths: 5- or 10-mm PEG-fusion groups and 5- and 10-mm NC groups. The 5-mm PEG-fusion group exhibited similar SFI scores to its paired NC group for 1–5 weeks PO (**Fig 1A-1C**). At 6 weeks PO, the 5-mm PEG-fusion group had significantly higher SFI scores than its paired NC group (−77 ± 6.5 vs. −111 ± 3.7; *p* < 0.0001, Tukey’s). Previous studies established an SFI score of −79 as the threshold for SFI recovery in PEG-fused rats following sciatic SL-PNIs [20,24,45]. Animals were classified as having successful behavioral recovery if they maintained SFI scores better than −79 starting at 6w PO and as having poor behavioral recovery if they failed to reach that threshold. Successful PEG-fused rats had significantly better SFI scores than NC rats at 6 weeks PO (PEG: −66 ± 6.2; *p* < 0.001, Tukey’s). The success rate of SFI recovery of the 5-mm PEG-fusion group was 67%, which was significantly higher (*p* < 0.05, Chi-square) than that of the 5-mm NC group at 0%.

The average SFI scores of all animals in the 10-mm PEG-fusion group were similar to the SFI scores of its paired NC group at all PO times (**Fig 1D-1F;** 6 weeks: PEG: −87 ± 7.6; NC: −92 ± 5.6; *p* > 0.05, Tukey’s). Successful PEG-fused rats had significantly better SFI scores than NC rats at 6 weeks PO (–67 ± 7.9 vs. –92 ± 5.6; *p* < 0.05, Tukey’s). One NC rat had successful SFI recovery at 6 weeks PO, a result occasionally seen in previous studies [24,46]. The success rate of SFI recovery in the 10-mm PEG-fusion group was 50%, which was not significantly different from the 67% success rate for the 5-mm PEG-fusion group (*p* > 0.05, Chi-square), but was significantly higher (*p* < 0.05, Chi-square) than the 11% success rate of the 10-mm NC group.

### PEG-fused VPNI repairs of SL-PNIs of different gap lengths produce similar recovery of axonal morphology

**Fig 2:**
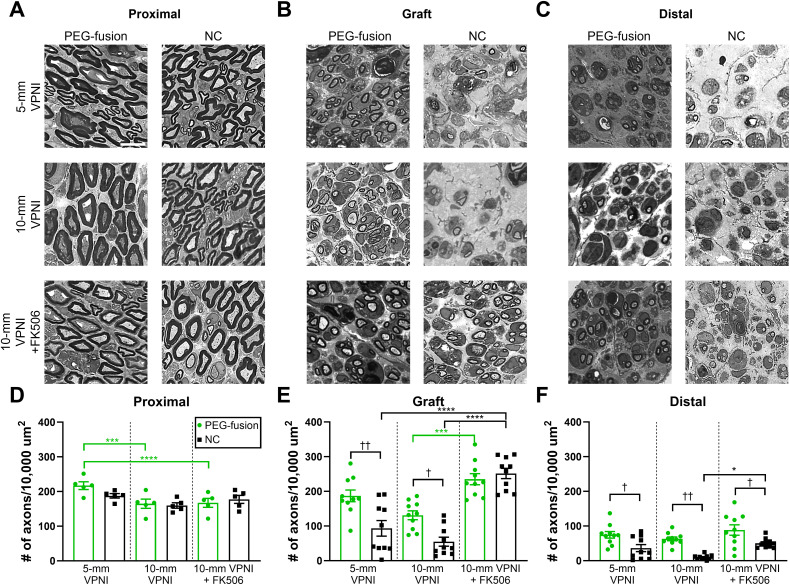
PEG-fusion repair using VPNIs preserved many axons by 3 weeks postoperatively, and localized FK506 promoted early axonal regeneration. **(A-C)** Representative (**A**) proximal, (**B**) mid-graft, and (**C**) distal thick sections (0.5µm). Scale bar = 10µm. **(D-F)** Quantification of axonal density in the (**D**) proximal, (**E**) mid-graft, and (**F**) distal nerve segments. One-way ANOVA statistics: For **D**, *F*_*(5,24)*_=3.81, *p* < 0.05; for **E**, *F*_*(5,54)*_=23.21, *p* < 0.0001; for **F**, *F*_*(5,54)*_=10.23, *p* < 0.0001. *s indicate Tukey’s multiple comparisons test results between PEG-fusion groups (green) or between NC groups (black). †s indicate Tukey’s multiple comparisons test results between PEG-fusion and NC groups. * or †: *p* < 0.05, ** or ††: *p* < 0.01, ***: *p* < 0.001, ****: *p* < 0.0001. *n* = 5-10 regions of interest (ROIs) pooled from 1-2 animals per group. Values are means ± SEM.

The axonal density at 3 weeks PO was analyzed for proximal, mid-VPNI, and distal segments of PEG-fusion and NC groups of 5- and 10-mm VPNIs using Tukey’s multiple comparison tests (**Fig 2A-2C**; **Table 1**). The 5-mm PEG-fusion group had significantly higher axonal densities than its paired NC group for both VPNIs (**Fig 2E**; 187 ± 17 vs. 93 ± 23; *p* < 0.01) and distal segments (**Fig 2F**; 75 ± 9.3 vs. 36 ± 10; *p* < 0.05). Similar differences in axonal densities were observed between the 10-mm PEG-fusion group and its paired NC group for VPNIs (**Fig 2E**; 131 ± 13 vs. 55 ± 13; *p* < 0.05) and distal segments (**Fig 2F**; 63 ± 5.5 vs. 10 ± 2.2; *p* < 0.01). These differences are due to surviving PEG-fused axons in PEG-fused VPNIs and distal segments that did not undergo WD. These surviving axons were absent in NC groups in which all axons underwent WD in VPNIs and distal segments [24]. Proximal segments (**Fig 2D**) of the 5-mm PEG-fusion group had a significantly higher axonal density than the 10-mm PEG-fusion group (217 ± 11 vs. 165 ± 13; *p* < 0.001). No significant differences were found for the VPNIs or distal segments between these two groups (**Fig 2E-2F;** *p* > 0.05).

**Table 1:** Descriptive summary of axon morphology.

Group	3w PO axon density (axons/10,000 µm^2^)			6w PO axon density (axons/10,000 µm^2^)			6w PO axon diameter (µm)		6w PO g-ratio	
	Proximal	Graft	Distal	Proximal	Graft	Distal	Graft	Distal	Graft	Distal
5-mm PEG-fusion	217 ± 11	187 ± 17	75 ± 9.3	211 ± 23	328 ± 40	276 ± 21	2.65 ± 0.07	2.10 ± 0.05	0.548 ± 0.005	0.558 ± 0.006
5-mm NC	188 ± 6.5	93 ± 23	36 ± 10	234 ± 16	329 ± 15	321 ± 23	1.72 ± 0.04	1.66 ± 0.04	0.600 ± 0.005	0.560 ± 0.004
10-mm PEG-fusion	165 ± 13	131 ± 13	63 ± 5.5	183 ± 23	401 ± 15	351 ± 29	2.13 ± 0.05	1.86 ± 0.04	0.586 ± 0.004	0.554 ± 0.004
10-mm NC	160 ± 7.8	55 ± 13	10 ± 2.2	187 ± 7.2	268 ± 17	262 ± 23	1.80 ± 0.04	1.55 ± 0.03	0.639 ± 0.004	0.614 ± 0.004
10-mm PEG-fusion+FK506	167 ± 13	235 ± 16	89 ± 15	123 ± 9.2	348 ± 26	315 ± 9.0	2.28 ± 0.09	2.04 ± 0.06	0.584 ± 0.008	0.539 ± 0.006
10-mm NC + FK506	177 ± 12	252 ± 15	50 ± 4.4	155 ± 11	307 ± 19	185 ± 14	1.76 ± 0.03	1.74 ± 0.03	0.631 ± 0.004	0.643 ± 0.004

**Fig 3:**
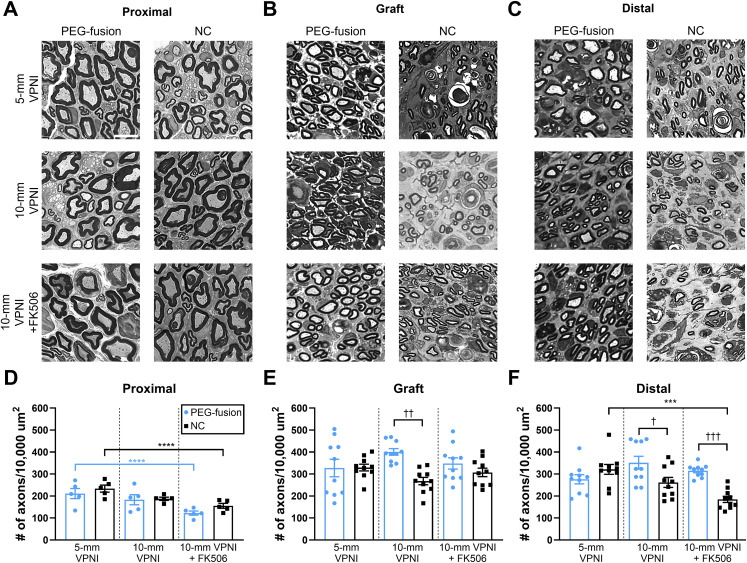
PEG-fusion preserved many axons by 6 weeks postoperatively, but early regenerative effect of localized FK506 diminished. **(A-C)** Representative (**A**) proximal, (**B**) mid-graft, and (**C**) distal thick sections (0.5µm). Scale bar = 10µm. **(D-F)** Quantification of axonal density in the (**D**) proximal, (**E**) mid-graft, and (**F**) distal nerve segments. One-way ANOVA statistics: For **D**, *F*_*(5,24)*_=5.87, *p* < 0.01; for **E**, *F*_*(5,54)*_= 3.461, *p* < 0.01; for **F**, *F*_*(5,54)*_= 7.957, *p* < 0.0001. *s indicate Tukey’s multiple comparisons test results between Successful PEG-fusion groups (blue) or between NC groups (black). †s indicate Tukey’s multiple comparisons test results between PEG-fusion and NC groups. †: *p* < 0.05, ††: *p* < 0.01, *** or †††: *p* < 0.001, ****: *p* < 0.0001. *n* = 5-10 ROIs pooled from 1-2 animals per group. Values are means ± SEM.

The axonal density at 6 weeks PO was analyzed as described above for 3 week PO segments (**Fig 3A-3C**; **Table 1**). No significant differences were found between the 5-mm PEG-fusion group and its paired NC group for any of the three nerve segments (**Fig 3D-3F;** *p* > 0.05). The 10-mm PEG-fusion group had significantly higher axonal densities than its paired NC group for VPNIs (**Fig 3E**; 401 ± 15 vs. 268 ± 17; *p* < 0.01) and the distal segments (**Fig 3F**; 351 ± 29 vs. 262 ± 23; *p* < 0.05). No significant differences in axonal density were found among any of the three nerve segments (**Fig 3D-3F;** *p* > 0.05) between the 5- and 10-mm PEG-fusion groups.

**Fig 4:**
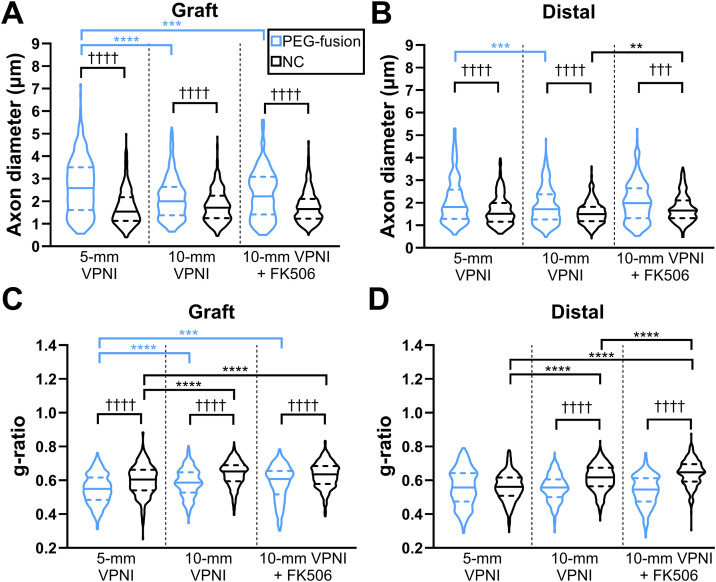
PEG-fusion maintained large-diameter, well-myelinated axons at 6 weeks PO. **(A-B)** Quantification of axonal diameter in the graft and distal nerve segments, respectively. One-way ANOVA statistics: For **A**, *F*_*(5,2036)*_=54.49, *p* < 0.0001; for **B**, *F*_*(5,1990)*_=27.13, *p* < 0.0001. **(C-D)** Quantification of g-ratio in the graft and distal nerve segments, respectively. One-way ANOVA statistics: For **C**, *F*_*(5,2036)*_=56.17, *p* < 0.0001; for **D**, *F*_*(5,1990)*_=72.22, *p* < 0.0001. *s indicate Tukey’s multiple comparisons test results between Successful PEG-fusion groups (blue) or between NC groups (black). †s indicate Tukey’s multiple comparisons test results between PEG-fusion and NC groups. **: *p* < 0.01, *** or †††: *p* < 0.001, **** or ††††: *p* < 0.0001. *n* = > 300 axons pooled from 1-2 animals per group.

The axonal diameter at 6 weeks PO of the 5-mm PEG-fusion group was significantly larger than its paired NC group for both VPNIs (**Fig 4A**; 2.65 ± 0.07 µm vs. 1.72 ± 0.04 µm; *p* < 0.0001) and distal segments (**Fig 4B**; 2.10 ± 0.05 µm vs. 1.66 ± 0.04 µm; *p* < 0.0001). The 5-mm PEG-fusion group also had a significantly lower g-ratio than its paired NC group (**Fig 4C**; 0.548 ± 0.005 vs. 0.600 ± 0.005; *p* < 0.0001). Similarly, the 10-mm PEG-fusion group had significantly larger axonal diameters (**Fig 4A-4B;** graft: 2.13 ± 0.05 µm vs. 1.80 ± 0.04 µm; *p* < 0.0001; distal: 1.86 ± 0.04 µm vs. 1.55 ± 0.03 µm; *p* < 0.0001) and lower g-ratios (**Fig 4C-4D;** graft: 0.586 ± 0.004 vs. 0.639 ± 0.004; *p* < 0.0001; distal: 0.554 ± 0.004 vs. 0.614 ± 0.004; *p* < 0.0001) than its paired NC group. The 10-mm PEG-fusion group had significantly lower axonal diameters (**Fig 4A-4B;** *p* < 0.001) and higher g-ratios (**Fig 4C**; *p* < 0.0001) than the 5-mm PEG-fusion group. The two NC groups had no significant differences (*p* > 0.05) in axonal diameter, but the 10-mm NC group had significantly higher g-ratios than the 5-mm NC group (**Fig 4C-4D;** *p* < 0.0001).

### Localized FK506 transiently improves axonal regeneration, but not behavioral recovery

In addition to the four groups analyzed above, two additional groups of rats were generated to investigate the effect of localized FK506 delivery. The surgical procedures for these two groups were the same as the previous PEG-fusion and NC groups repaired using 10-mm VPNIs, except that FK506 contained in TISSEEL fibrin gel was delivered locally after nerve repair and before wound closure. The 10-mm PEG-fusion group treated with FK506 had similar average SFI scores compared to its paired NC group at all PO times (**Fig 1G-1I**; 6 weeks: PEG: −99.3 ± 9.6; NC: −99.7 ± 5.6; *p* > 0.05, Tukey’s). Only one rat in the PEG-fusion group treated with FK506 achieved successful SFI recovery, i.e., a success rate of 8% for this group. Two of 11 NC rats treated with FK506 also achieved recovery by 6 weeks PO, an 18% success rate. These recovery rates were not significantly different from each other (*p* > 0.05, Chi-square) and were both significantly lower (*p* < 0.05, Chi-square) than the rates (67% and 50%) for the 5- and 10-mm PEG-fusion groups without FK506.

The axonal density at 3 weeks PO was analyzed for proximal, VPNI, and distal nerve segments of PEG-fusion and NC groups with and without FK506 using Tukey’s multiple comparison tests (**Fig 2A-2C**; **Table 1**). FK506 treatment had no effect in the PEG-fusion or the NC group (*p* > 0.05) for proximal segments (**Fig 2D**). FK506 treatment significantly increased axonal density for the 10-mm PEG-fusion group for VPNIs (**Fig 2E**: 235 ± 16 vs. 131 ± 13; *p* < 0.001). FK506 treatment also significantly increased axonal density for the 10-mm NC group compared to the 10-mm NC group without FK506 (252 ± 15 vs. 55 ± 13; *p* < 0.0001) and the 5-mm NC group (93 ± 23; *p* < 0.0001). For distal segments (**Fig 2F**), FK506 treatment significantly increased axonal density for the 10-mm NC group (50 ± 4.4 vs. 10 ± 2.2; *p* < 0.05). The PEG-fusion group with FK506 exhibited higher axonal density than the NC group with FK506 (89 ± 15 vs. 50 ± 4.4; *p* < 0.05). These findings suggest that localized FK506 treatment increases axonal density at 3 weeks PO.

The axonal density at 6 weeks PO with regards to FK506 was analyzed as described above for 3 week PO segments (**Fig 3A-3C**; **Table 1**). For all nerve segments (**Fig 3D-3F**), no significant difference (*p* > 0.05) was found between the 10-mm PEG-fusion groups with or without FK506 or between the 10-mm NC groups with or without FK506. The 10-mm NC group with FK506 had significantly lower axonal density than the 10-mm PEG-fusion group with FK506 (185 ± 14 vs. 315 ± 9.0; *p* < 0.001) for the distal segments (**Fig 3F**). These results show that localized FK506 treatment did not increase axonal density at 6 weeks PO.

Axonal diameter at 6 weeks PO for the 10-mm PEG-fusion group with FK506 was significantly larger than its paired NC group for both VPNIs (**Fig 4A**; 2.28 ± 0.09 µm vs. 1.76 ± 0.03 µm; *p* < 0.0001) and distal segments (**Fig 4B**; 2.04 ± 0.06 µm vs. 1.74 ± 0.03 µm; *p* < 0.001). The 10-mm PEG-fusion group with FK506 also had significantly lower g-ratios than its paired NC group for VPNIs (**Fig 4C**; 0.584 ± 0.008 vs. 0.631 ± 0.003; *p* < 0.0001) and distal segments (**Fig 4D**; 0.539 ± 0.006 vs. 0.643 ± 0.004; *p* < 0.0001). No significant differences in axonal diameter or g-ratio (**Fig 4**; *p* > 0.05) were found for the 10-mm PEG-fusion groups with and without FK506. In contrast, FK506 treatment significantly increased distal axonal diameter (**Fig 4B**; 1.74 ± 0.03 µm vs. 1.55 ± 0.03 µm; *p* < 0.01) and g-ratio (**Fig 4D**; 0.643 ± 0.004 vs. 0.614 ± 0.004; *p* < 0.0001) for the 10-mm NC group.

In summary, localized FK506 treatment increased axonal density at 3 weeks PO for both PEG-fusion and NC repairs. However, it did not maintain these effects, as the treatment did not alter axonal diameter or g-ratio for PEG-fusion repairs at 6 weeks PO and produced poorer SFI recovery, the most important measure of successful repair.

## Discussion

### Effects of PEG-fusion of VPNIs, gap length, and FK506 on behavioral recovery after SL-PNIs in rats

**Fig 5:**
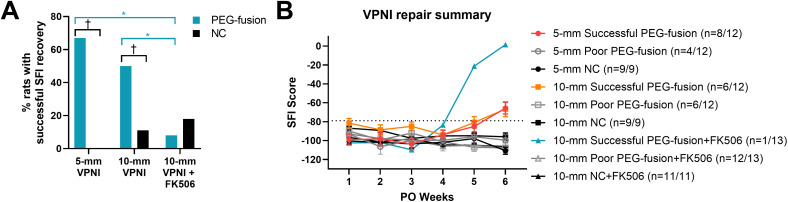
SFI recovery summary of PEG-fused VPNI repairs. Successful and poor SFI recovery were defined as maintaining SFI score of>-79 or failing to reach SFI score of −79 by 6 weeks PO, respectively. **(A)** SFI recovery success rate. *s indicate Chi-square test results between PEG-fusion groups (blue). †s indicate Tukey’s multiple comparisons test results between PEG-fusion and NC groups. * or †: *p* < 0.05. **(B)** SFI recovery of all PEG-fusion groups separated by recovery success plotted against NC groups. Note that successful SFI recovery rate in PEG-fusion+FK506 group was low and comparable to those of negative control groups despite the excellent SFI score of the only successful PEG-fusion+FK506 rat.

**Fig 5** summarizes the SFI recovery of 5- and 10-mm VPNI repairs with and without FK506 treatment. PEG-fusion repairs of 4- and 8-mm long SL-PNIs produced similar successful SFI behavioral recovery rates (**Fig 5A**, 67% and 50%), both significantly better than their paired neurorrhaphy-only NC repairs (0% and 11%). One rat in the FK506 10-mm PEG-fusion group had an excellent SFI score (**Fig 5B**). However, localized FK506 treatment significantly decreased the average SFI behavioral recovery rate for the 10-mm PEG-fusion group from 50% to 8% (**Fig 5A**), a rate similar to neurorrhaphy-only NCs. Localized FK506 treatment did not significantly increase the SFI behavioral recovery rate (from 11% to 18%) for the FK506 10-mm NC group that had no fused axons and relied solely on axonal regeneration.

### PEG-fusion repair of shorter and longer SL-PNIs using appropriately sized VPNIs

Previous studies of PEG-fusion repair using viable autografts [24,26] or viable allografts [6,19,24,45,47–49] did not strictly control graft length, which often ranged between 5–10 mm, as an experimental variable that might affect behavioral recovery and other outcomes. Nonetheless, in these studies using various graft lengths, immediate electrophysiological continuity was consistently restored, many axons did not undergo WD, and successful behavioral recovery as assessed by the SFI test was observed in most animals in the PEG-fusion groups. The current study is the first to control graft length as a variable in the context of PEG-fusion repair of SL-PNIs using VPNIs. We used VPNIs instead of allografts in this study to eliminate immunological rejection as a confounding variable. Following 4- and 8-mm-long SL-PNIs, 5- and 10-mm PEG-fused VPNI repairs successfully preserved many axons and produced successful SFI recovery. In contrast, their paired NC groups did not successfully maintain axons or produce successful SFI recovery.

Although the 10-mm PEG-fusion group produced significantly lower average axonal diameter and higher g-ratio than the 5-mm PEG-fusion group at 6 weeks PO, the axonal densities were not significantly different between the two groups. These results suggest that 10-mm PEG-fused VPNIs contained fewer well-myelinated PEG-fused axons than 5-mm PEG-fused VPNIs. However, as noted above, both shorter (5 mm) and longer (10 mm) PEG-fused VPNI repairs produced similar SFI behavioral recovery rates. Our current study supports previous hypotheses [49] that behavioral recovery can be achieved following PEG-fusion if there are a sufficient number of successfully fused axons.

### Effects of localized application of FK506 in TISSEEL

FK506 reduces rejection of peripheral nerve allografts *that are not PEG-fused* [27,28,50,51] and is a potent neuromodulator for axonal regeneration when used locally [34–39,52–55]. Our study eliminated allograft rejection as a confounding variable by using VPNIs. PEG-fusion produces two axonal populations in VPNIs—successfully fused axons that are maintained long-term and unsuccessfully fused axons that undergo WD and then regeneration [6,20,24,25,45]. We hypothesized that localized FK506 would promote axonal regeneration and would not impair the ability to successfully fuse axons.

Our results show that localized FK506 did not alter the diameter or g-ratio of surviving PEG-fused axons at 6 weeks PO and increased axonal density in the VPNIs for both PEG-fusion and NC repair groups at 3 weeks PO. However, localized FK506 treatment did not significantly affect axonal densities at 6 weeks PO in either the PEG-fusion or NC VPNI groups. More importantly, FK506 treatment produced poorer behavioral recovery at 6 weeks PO in rats with PEG-fused VPNIs. It is possible that FK506 concentration decreased by 6 weeks PO, as reported by previous studies that used similar dosages and timing to ours [36,53,54]. Therefore, the lack of long-term effects of localized FK506 treatment may limit its clinical applicability, as natural axonal regeneration in humans typically takes months to years, during which distal muscles undergo atrophy and may suffer permanent functional loss [4,6].

### Limitations

In this study, we examined VPNIs of two different lengths (5- and 10-mm) in rats. Grafts of an even longer length in larger animal models that may be more clinically relevant should be included in future studies to validate our findings. We also note that successful SFI recovery rates of PEG-fused allografts in other publications using rats (60–90%: [24,45,47,48]) appear similar or slightly better than those of PEG-fused VPNIs (50–67%) in the current study. Therefore, future studies should address whether the efficacy of PEG-fusion differs between allografts and VPNIs. Furthermore, whether histological and behavioral outcomes following PEG-fusion of allografts of different lengths are similar should be validated. Finally, FK506 results in this study were limited to one specific dose and delivery method. Future studies may explore whether the efficacy of local FK506 delivery in rats improves with higher doses or better delivery methods that extend the release window. FK506 results in smaller animal models like rats need to then be examined in larger animal models like swine to translate to best clinical practices.

## Conclusions

In brief, behavioral recovery remains the most meaningful measure of outcome following traumatic SL-PNIs. In this study, repair of shorter or longer SL-PNIs using PEG-fused VPNIs produced comparable behavioral recoveries in rats. Future studies need to demonstrate that PEG-fusion repairs using allografts of shorter and longer graft lengths in larger animal models produce similar data to our isograft studies. Such data would further support the translation of PEG-fusion repair towards clinical uses.

## Supporting information

S1 File Axon morphology.pzfx. Axon morphology raw data.
(PZFX)

S2 File Axon morphology.xlsx. Axon morphology raw data.
(XLSX)

S3 File SFI summary.pzfx. SFI raw data.
(PZFX)

S4 File SFI summary.xlsx. SFI raw data.
(XLSX)
